# Stress and Coping in Teens with Chronic Physical Health Conditions: A Cross-Sectional Study

**DOI:** 10.3390/children12070858

**Published:** 2025-06-28

**Authors:** Anne L. Ersig, Rachel Hawn, Niamh Nolan, Roger L. Brown

**Affiliations:** 1 University of Wisconsin-Madison School of Nursing, 701 Highland Ave, Madison, WI 53705, USA; rhawn@wisc.edu (R.H.);; 2 UW Health Kids, Madison, WI 53792, USA

**Keywords:** stress, coping, adolescent, chronic health condition

## Abstract

Background/Objectives: Adolescents with chronic physical health conditions (CHCs) use specific coping strategies to respond to condition-related stressors. However, most studies of CHC-related stress and coping focus on a single condition. The objective of this study was to measure CHC-related stress and identify associated coping strategies in adolescents with a variety of CHCs. A secondary objective was to examine the relationship between CHC-related stress, coping strategies, health-related quality of life, and perceived severity of chronic illness. Methods: Teens (*n* = 38, 68.42% female, mean age 17.9 years) with CHCs completed the Responses to Stress Questionnaire (RSQ) for CHC-related stress, the PedsQL to assess health-related quality of life, and the Perceptions of the Severity of Chronic Illness (PSCI) measure. The most frequently reported conditions were asthma, food allergies, and multiple conditions. We used fuzzy cluster analysis to identify two clusters, high stress and low stress, based on ratings of CHC-related stressors. Relationships between coping strategies and the PedsQL and PSCI, and between the PSCI and PedsQL, were assessed using Pearson partial correlations. Relationships between the PSCI, PedsQL, and coping strategies for the two clusters were assessed using adjusted mean differences. We adjusted for multiple comparisons by controlling the false discovery rate. Significance was set at *p* < 0.05. Results: Teens were most likely to use secondary control engagement coping and involuntary engagement to respond to CHC-related stressors. Teens in the two clusters differed on health-related quality of life but not coping strategies or perceived condition severity. CHC diagnosis category was associated with cluster membership. Conclusions: This exploratory study highlighted relationships among quality of life, coping strategies, and CHC diagnosis category that should be explored in future studies. Improved understanding of CHC-related stress and coping strategies in teens with CHCs could have an impact on their quality of life and well-being.

## 1. Introduction

The prevalence of childhood-onset chronic health conditions in the United States (US) is increasing. Up to 20% of children and adolescents in the US have a diagnosed chronic health condition (CHC) [1]. CHCs such as diabetes, asthma, or food allergies last longer than acute illnesses and many have an impact on daily life, increasing the risk of experiencing condition-related acute and chronic stress [2,3,4,5]. Adolescence is also a critical developmental phase with a high rate of general life stress exposures. As a result, adolescents with CHCs experience general and CHC-related stress exposures [5,6,7,8,9,10] and high levels of perceived stress [6,11,12,13,14].

How adolescents with CHCs respond to CHC-related stressors has important implications for adaptation and adjustment to life with a CHC [5]. Individual stress responses include automatic bodily reactions as well as directed, voluntary coping strategies [5,15]. Individual coping strategies also involve engagement or disengagement. Engagement coping responses are directed towards the stressor, while disengagement coping strategies are used to avoid the stressor [15]. In this study, adolescent coping is defined as “…a collection of purposeful volitional efforts that are directed at the regulation of aspects of the self and the environment under stress” [5].

Different coping strategies are used to respond to stressors with different characteristics, and accurate assessment requires identifying coping strategies associated with specific stressors [5,15]. However, our understanding of CHC-related stress and coping in teens with a variety of CHCs is limited. Studies have focused on stress and coping related to specific diagnoses [16,17,18,19,20,21] or assessed general, not CHC-related, stress and coping [9,14,22]. The Europe-based DISABKIDS project developed a measure of coping strategies for children and adolescents with various chronic conditions [23]. However, this measure does not assess CHC-related stress and cannot be used to assess and compare CHC-related stress and associated coping strategies in teens with a variety of CHCs. The noncategorical approach to CHCs [24] highlights the common characteristics of chronic health conditions affecting youth. Consistent with this approach, assessing coping strategies for CHC-related stress in adolescents with chronic conditions will provide relevant information that could be used to develop interventions to facilitate adaptive coping across conditions [25].

The Responses to Stress Questionnaire (RSQ) is a validated measure of adolescent stress and coping strategies that was designed to be adapted for different stressors, providing insights into coping strategies used for specific stressors [5,15]. The measure is based on a model that includes voluntary and involuntary responses to stress and whether those responses involve engagement or disengagement from the stressor [5,15]. The RSQ identifies respondents’ use of five different coping strategies: primary control engagement coping, secondary control engagement coping, disengagement coping, and involuntary engagement and disengagement. These coping strategies are defined in Table 1, and examples of associated actions for each coping strategy are provided. Results from the RSQ provide proportions for the five coping strategies that reflect how frequently an individual uses each [15]. Each version of the RSQ lists 12 to 13 stressors specific to the domain or situation of interest. Respondents indicate how stressful each item has been over the last 6 months on a 1–4 Likert scale, as well as how much control they think they have over the stressors, then answer a series of questions on how they cope with those stressors. Items assessing teens’ coping strategies are consistent across versions of the RSQ, which has adequate to excellent reliability and validity across different domains of stress [5,15].

Versions of the RSQ have been developed to assess coping strategies for stress related to specific CHCs, such as diabetes, chronic pain, epilepsy, and sickle cell disease [27]. However, condition-specific measures of CHC-related stress and coping limit opportunities to understand shared experiences across conditions [28]. There are few studies of CHC-related stress and associated coping strategies in adolescents with a variety of chronic health conditions, even though teens with different CHCs may have similar stressors and strategies for coping with them [29]. Thus, we created a version of the RSQ to measure CHC-related stress and associated coping strategies in adolescents with a variety of CHCs. We were also interested in whether CHC-related stress and coping strategies differed according to teens’ quality of life or their perceptions of the severity of their CHCs. The RSQ for CHC-related stress we developed is available on the RSQ website under the name “Pediatric chronic health condition (general)” [27].

### Study Objective

The primary objective of this pilot study was to measure CHC-related stress and identify associated coping strategies in 14–18-year-old adolescents with a variety of chronic physical health conditions. Our secondary objective was to examine the relationship between CHC-related stress, coping strategies, and measures of health-related quality of life and perceived severity of chronic illness. As this was an exploratory pilot study, there were no testable hypotheses. However, based on results from previous studies [3], we sought to determine whether factors such as perceived severity of chronic illness were related to CHC-related stress and coping.

## 2. Materials and Methods

### 2.1. Participants

The population for this study was adolescents aged 14–18 years old who self-reported a diagnosis of a chronic physical health condition at least 12 months ago, were able to read and speak English, and were able to access an online survey. Chronic physical health conditions were defined as conditions that last longer than acute illnesses and are likely to have an impact on daily life, such as diabetes, asthma, or food allergies. We excluded teens who reported having developmental, behavioral, or cognitive disabilities, or who had a chronic physical health condition diagnosed in the last year. As this was a pilot study, we did not complete an a priori power analysis. For this study, the critical outcome is an estimate of the standard deviation of the effect to guide future studies [30]. The study received ethical approval from the University of Wisconsin-Madison Minimal Risk IRB (IRB# 2021-0147).

All recruitment materials included brief study information and a link to the study survey. Survey data were collected and managed using REDCap electronic data capture tools hosted at the University of Wisconsin-Madison School of Medicine and Public Health [31,32]. Recruitment methods included mass emails to a university ListServ, posts on public websites or in online support groups for parents of children with CHCs, and information shared through local family-focused email newsletters. The first page of the survey had information on the study, study activities, and IRB-approved elements of consent. Those who continued past the landing page verified that they gave parental permission and teen assent to participate for teens under 18, or teen consent to participate for those who were 18. Due to the open nature of recruitment, study team members carefully reviewed responses to identify those that were invalid. These included responses that were completed too quickly for the number of surveys and items in the study; had missing data; were completed in languages other than English; or had invalid responses for age of the teen. The final dataset included 38 teens with CHCs with complete, valid responses to all study measures, who received an electronic gift card for their time.

Study measures included demographics, the RSQ for CHC-related stress, the self-report version of the PedsQL 4.0 for Teens [33], and a measure of Perceived Severity of Chronic Illness (PSCI) [34].

For the RSQ for CHC-related stress, we generated a list of twelve CHC-related stressors common across conditions based on a review of the literature and measures of CHC-related stress for specific conditions. Previous studies identified CHC-related stressors experienced by adolescents with different CHCs, such as the intrusiveness of the condition, interruptions to daily routines, missing school or activities for medical appointments, condition-related crises, the impact on teens’ social lives and interactions, and the need to be constantly vigilant [2,3,5,35]. CHC-specific stress measures reviewed were the Diabetes Stress Questionnaire (DSQ) [6], questions used by the first author to assess food allergy-related stress in previous studies [3], and versions of the RSQ for specific CHCs (e.g., cystic fibrosis, type 1 diabetes, inflammatory bowel disease) [27]. Concepts for stressors common across CHCs were identified and used to generate the list of 12 stressors (Supplementary Materials, Box S1). Participants provided ratings for how stressful each stressor was over the last 6 months on a 4-point Likert scale (1 = not at all, 2 = a little, 3 = somewhat, 4 = very), then completed the remaining questions assessing coping strategies for CHC-related stress. Scoring for the RSQ generated proportions reflecting how frequently individuals used primary control engagement coping, secondary control engagement coping, disengagement coping, and involuntary engagement and disengagement [15].

Health-related quality of life was assessed using the PedsQL 4.0 Teen Self Report module. The PedsQL is a modular measure of health-related quality of life (HRQOL) for children and adolescents. It includes generic core scales as well as those that are specific to certain conditions (https://www.pedsql.org (accessed on 29 December 2024)). For this study, we used the Teen Self Report Generic Core scales. Results include the total score for overall HRQOL as well as physical, emotional, social, and school functioning. The psychosocial health summary score reflects emotional, social, and school functioning [33,36,37]. Scale scores are calculated as means, using the sum of the items over the number of items answered, which accounts for missing data. Higher scores indicate higher HRQOL. The PedsQL Teen Self Report module has been used extensively with healthy and patient populations and has established reliability (Cronbach’s α range, 0.87–0.92) and validity in teens with and without chronic health conditions [33,38].

The Perceived Severity of Chronic Illness (PSCI) measure developed by Leung and colleagues [34] was used to assess adolescents’ perceptions of the severity of their chronic illness. The 14-item scale consists of a series of statements about individual perceptions of a chronic illness (e.g., I look different to people my own age). Areas addressed include body image, perceived seriousness of the condition, and its effects on activity, social life, academics, future expectations, reproduction, and relationships with parents. The PSCI uses a five-point Likert scale assessing agreement with a statement about the chronic illness (agree completely, agree, unsure, disagree, or disagree completely). Eight items are reverse scored and higher total scores reflect higher perceived severity (range, 14–70). Reliability and validity of the PSCI have not been formally assessed, but it has been used in other studies of adolescents and young adults with chronic health conditions [3,39].

### 2.2. Statistical Methods

Data were analyzed using NCSS Version 19 [40]. All analyses controlled for age, biological sex assigned at birth, and CHC diagnosis category. Descriptive data include frequencies and means as appropriate. Fuzzy clustering using the Manhattan distance was used to group participants into clusters, using their ratings of the 12 stressors assessed in the RSQ for CHC-related stress. Fuzzy clustering provides graded cluster assignment for identification of clear group members vs. those near cluster boundaries. Fuzzy clustering using Manhattan distance is a soft clustering method that calculates similarity using the sum of absolute differences, and assigns each data point a probability of membership in each cluster [41]. To evaluate which clustering solution is optimal, we compared key validity metrics across 2-, 3-, and 4-cluster solutions, including the average silhouette coefficient and fuzzy clustering indices of Dunn’s partition coefficient (F(U), and Dunn’s normalized coefficient (Fc(U) [42], along with Kaufman and Rousseeuw’s (1990) partition coefficient (D(U) and normalized coefficient (Dc(U) [43], for each possible solution. Selection of the appropriate number of clusters is based on maximizing the average silhouette coefficient and Fc(U) while minimizing Dc(U). In this case, a two-cluster solution was selected, which identified a high-stress cluster and a low-stress cluster. Parametric Pearson partial correlations were used to assess relationships between RSQ coping strategies and the PedsQL and PSCI as well as between the PSCI and PedsQL, an approach selected so that we could control for the covariates of biological sex, age, and CHC diagnosis category. Relationships between the PSCI, PedsQL, and coping strategies across the two stress-related clusters were assessed using adjusted mean differences and tested using the Wald test statistic. We adjusted for multiple comparisons by controlling for the false discovery rate (FDR) using the Benjamini–Hochberg FDR [44]. We used the 70% rule for missing data. If a scale had 30% or less missing data, it was assumed to be missing at random, and the means were calculated on the available data instead of imputing the missing values. Significance for all analyses was set at *p* < 0.05 to identify relationships of interest and guide future research.

## 3. Results

### 3.1. Study Sample

The study sample includes 38 14–18-year-old adolescents diagnosed with a chronic physical health condition at least 12 months prior to study enrollment. Teens were, on average, 17.9 years old (range, 15–18 years). Most teens identified as white (78.95%) and were assigned female at birth (68.42%; Table 2). Teens reported a variety of CHCs, including asthma, food allergies, ulcerative colitis, and type 1 diabetes. Several teens had multiple CHCs (e.g., asthma and food allergies).

### 3.2. Fuzzy Cluster Analysis of Stressor Ratings

**Figure 1:**
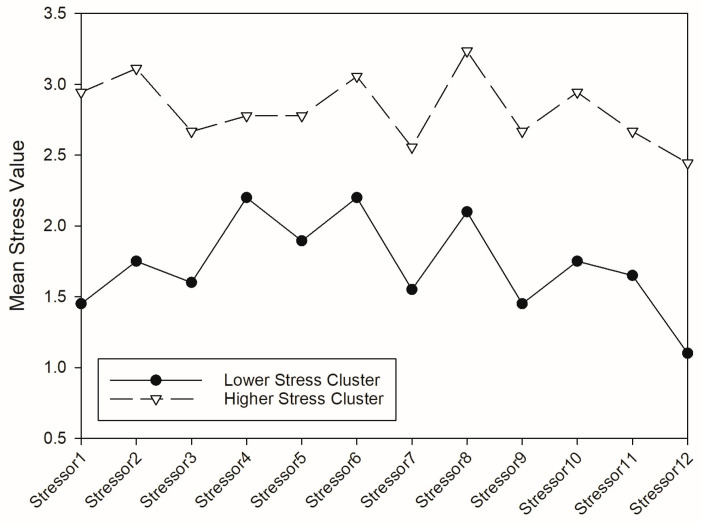
Clusters were identified using fuzzy cluster analysis.

Fuzzy cluster analysis of teens’ ratings for the 12 stressors on the RSQ for CHC-related stress identified two clusters, a high-stress cluster (*n* = 18) with higher scores for all 12 stressors and a low-stress cluster (*n* = 20) (Figure 1; Supplementary Material, Table S1). Cluster selection was based on a comparison of key validity metrics, including the average silhouette coefficient and fuzzy clustering indices F(U), Fc(U), D(U), and Dc(U), for two-, three-, and four-cluster solutions (Table 3) [41]. Selection of the appropriate number of clusters was based on maximizing the average silhouette coefficient and Fc(U) while minimizing Dc(U).

We then explored whether the CHC diagnosis category was related to cluster membership. We grouped unique conditions reported by small numbers of teens into three categories, GI conditions, multiple conditions affecting different body systems, and other conditions. For more common conditions (i.e., asthma, food allergy, type 1 diabetes), we used the reported CHC diagnosis. We then examined cluster membership according to CHC category (Supplementary Materials, Table S2). We found that CHC diagnosis categories were related to cluster membership. For some categories of teens with a specific diagnosis (e.g., food allergies, type 1 diabetes), all teens were in one cluster. For other categories that included teens with different diagnoses, teens were in both clusters. Teens with asthma were in both categories.

Additional analyses compared gender, biological sex assigned at birth, and age between clusters (Table 4). There were no differences in gender, biological sex, or age between clusters.

### 3.3. Descriptive Findings

Study participants were most likely to use secondary control engagement coping (mean proportion, 0.26; Table 5). Involuntary engagement was the second most common coping strategy (mean proportion, 0.24). Participants were least likely to use primary control engagement coping (mean proportion, 0.17) and disengagement coping (mean proportion, 0.15). The mean total score for the PedsQL was 73.42 (SD 19.42), with subscale means from 62.73 (SD 24.05) for emotional functioning to 82.97 (SD 18.79) for social functioning. The subscale for physical functioning had a mean score of 74.49 (SD 20.99), with a range of 21.87 to 100. Scores for the PSCI had a mean of 32 (SD 8.98, range 16–52; Table 5).

### 3.4. Relationships Between Measures

PedsQL total and subscale scores were negatively associated with PSCI scores, indicating that teens who thought of their CHC as more severe had a lower HRQOL (all adjusted *p* < 0.05).

We used parametric Pearson partial correlations to examine relationships between coping strategies and the PedsQL Total Score and PSCI, controlling for age, biological sex, and CHC diagnosis category (Table 6). Results showed that the PedsQL total score was positively associated with primary and secondary control engagement coping and negatively associated with disengagement coping, involuntary engagement, and involuntary disengagement. The results for the association between the perceived severity of chronic illness and secondary control engagement coping did not reach conventional statistical significance (*p* = 0.065), but suggest a possible association worthy of further study. The perceived severity of the chronic illness was not significantly associated with any of the other coping strategies.

Adjusted mean differences between cluster membership and coping strategies were analyzed using the Wald test (Table 7). There were no significant differences in coping strategies between the high- and low-stress clusters. Although results did not reach statistical significance, teens in the high-stress cluster were slightly more likely to use involuntary engagement coping (*p* = 0.06). We also analyzed differences in quality of life and perceived severity of the chronic illness between clusters (Table 7). Teens in the low-stress cluster had significantly higher scores for the PedsQL total score and all PedsQL subscales. Differences between clusters for perceived severity of the chronic illness trended towards significance (mean ± SE: 36.25 ± 2.25 vs. 28.60 ± 2.08, *p* = 0.063).

### 3.5. Feedback on the RSQ for CHC-Related Stress

Approximately 63% of adolescents said it was easy or very easy to complete the RSQ (*n* = 24), 31% said it was neither easy nor difficult (*n* = 13), and only two teens replied that it was difficult (5.2%). Teens’ reasons for their answers were not assessed.

## 4. Discussion

The objectives of this exploratory, cross-sectional study were to measure CHC-related stress and identify associated coping strategies in adolescents with a variety of CHCs. We also examined the relationship between CHC-related stress, coping strategies, health-related quality of life, and perceived severity of chronic illness. We identified secondary control engagement coping and involuntary engagement as the most frequently used coping strategies for teens with CHCs. We also identified two clusters of teens, a high-stress cluster and a low-stress cluster, and found that the CHC diagnosis category was associated with cluster membership. There were no significant differences between clusters for coping strategies or perceived severity of chronic illness. Teens in the two clusters did differ on HRQOL. While the results of this study are highly exploratory, they suggest directions for future research on CHC-related stress and coping in teens with a variety of chronic health conditions.

The most frequently used coping strategies in this study of teens with different CHCs aligned with those from previous studies of coping strategies in teens with specific CHCs. We found that teens were most likely to use secondary control engagement coping and involuntary engagement, and least likely to use primary control engagement coping and disengagement coping. These results align with those in studies of teens with type 1 diabetes [26], sickle cell anemia [46], and congenital heart disease [47]. While most CHCs can be managed, they can also be unpredictable and difficult to control [5,48,49]. Secondary control coping is an adaptive response; those who choose this strategy are adapting or adjusting to their situation, without trying to change it [50]. Teens who use coping strategies associated with secondary control engagement coping, such as positive thinking, cognitive restructuring, acceptance, and distraction, may be choosing to adapt to situations causing stress, rather than trying to influence them [5,15,26]. Additional studies are needed to explore whether specific CHC-related stressors, or types of stressors, are associated with particular coping strategies, teens’ reasons for selecting coping strategies, and whether they perceive those strategies as helpful.

Secondary control engagement coping and the PedsQL total score were positively associated, similar to findings from studies of teens with type 1 diabetes and sickle cell disease [44,51]. In other studies, secondary control engagement coping in teens with CHCs has been shown to be negatively associated with depression and anxiety, which reflect lower HRQOL [47,51,52]. However, because this was a cross-sectional study, we are unable to determine whether use of adaptive coping strategies led to higher quality of life, or whether higher quality of life prompts teens to use adaptive coping strategies. Future longitudinal studies are needed to assess causality and determine whether teens with higher quality of life are more likely to choose adaptive coping strategies, or whether the use of adaptive coping strategies leads to higher quality of life.

Involuntary engagement, which includes responses such as physiological arousal and rumination, was the second most common coping strategy identified in this study. These stress responses tend to be more automatic and habitual, and are associated with increased risk of depression and anxiety [15,47,53]. In this study, use of involuntary engagement was associated with lower total QOL. In other studies, involuntary engagement responses were associated with poor emotional adjustment in children and adolescents with recurrent abdominal pain [54] and lower quality of life in those with chronic pain [17]. Future studies are needed to explore these relationships in more depth. Assessing relationships between coping strategies and emotional adjustment or related concepts, such as anxiety or depressive symptoms, could provide more insights into relationships between involuntary engagement coping and other factors.

The high-stress and low-stress clusters were identified based on teens’ ratings of 12 CHC-related stressors. Coping strategies did not differ between the clusters. Future studies should explore what other factors, such as CHC characteristics, may be related to teens’ coping strategies. Our analyses did find significant differences in quality of life between the clusters, with teens in the low-stress cluster having higher quality of life in all domains.

As this is a cross-sectional study, future longitudinal studies with larger samples are needed to explore potentially complex relationships among CHC-related stressors, coping strategies, CHC characteristics, and quality of life. Other studies have found that teens with higher quality of life may be more likely to use adaptive coping strategies such as positive thinking and acceptance [15], and teens with chronic conditions have highlighted the importance of reframing and positive thinking due to their inability to change their situations [55]. Future qualitative and mixed-methods studies could explore whether these findings are influenced by diagnosis or other illness characteristics.

We also explored whether the CHC diagnosis category was related to cluster membership. We found that CHC diagnosis categories were associated with membership in clusters based on mean ratings of CHC-related stressors. This may indicate that specific diagnoses lead to higher stress than others. However, other CHC characteristics, such as visibility and manageability, may also contribute to CHC-related stress [56,57,58]. Future studies are needed that will explore how these other characteristics may be related to CHC-related stress and coping.

Findings from this study have implications for clinical practice and research. Due to its length, the RSQ for CHC-related stress would be challenging to implement in a clinical setting. However, clinicians in all settings who work with teens with CHCs should be aware of the impact that condition-related stress can have on teens. Assessing stressors that are common across CHCs (e.g., challenges related to management and accessing healthcare) would provide opportunities to share resources with teens and their families. Evidence also supports the integration of mental and physical healthcare for youth with CHCs [59,60,61]; based on this, clinicians could discuss the importance of mental healthcare with patients, which could lead to reduced stress and improved quality of life. Clinicians could also consider implementing evidence-based practices, such as those developed for teens with type 1 diabetes [18], to help teens with CHCs develop more adaptive strategies for coping with CHC-related stress. In addition, future research could explore the development of a shorter version of the RSQ for use in clinical settings.

Future studies are needed to explore the direction of the relationships between CHC-related stress and coping, including whether adolescents with CHCs use different coping strategies depending on their current sources of stress and the characteristics of those stressors (e.g., controllability). Prospective and longitudinal studies of stress exposure and response could provide insights into the influence of individual characteristics (e.g., tolerance for uncertainty, illness identity, illness acceptance), condition characteristics (e.g., visibility, uncertainty), and contextual factors (e.g., location, social determinants of health) on strategies. Future studies should also incorporate the RSQ for CHC-related stress and examine the relationship between coping strategies and additional measures of mental health, such as anxiety and depressive symptoms. These studies could help lay the foundation for improving quality of life and well-being for all teens with chronic health conditions.

The study reported here has several notable limitations. First, and most important, the study sample was small and homogeneous, which significantly limits the generalizability of the findings. To determine whether the RSQ for CHC-related stress is an appropriate measure for more diverse groups, future studies should use the RSQ for CHC-related stress with larger and more diverse samples to explore similarities and differences in stress exposure and response. The study was also cross-sectional, so we cannot draw conclusions about the direction of relationships between study variables. Larger longitudinal studies with more diverse samples are critical for advancing our knowledge on CHC-related stress and coping in teens with different CHCs. Future studies should also examine whether the list of stressors in the RSQ for CHC-related stress is sufficiently comprehensive. Teens were asked for suggestions for stressors to consider adding to the measure; however, there was significant variability in the open-text responses that precluded drawing definitive conclusions. A future study should incorporate a more structured approach to assessing teens’ suggestions for additional stressors.

## 5. Conclusions

Findings from this exploratory, cross-sectional study of CHC-related stress and coping strategies in teens with a variety of chronic conditions aligned with findings from studies of teens with specific CHCs. Prospective and longitudinal studies with larger sample sizes are needed to assess relationships among CHC-related stress, coping strategies, and quality of life in teens with different chronic health conditions.

## Figures and Tables

**Table 1:** Coping strategies, definitions, and associated actions.

Coping Strategy	Definition [26]	Associated Actions
Primary Control Engagement Coping	Attempts to influence objective events or conditions	Problem solving Emotional expression Emotional modulation
Secondary Control Engagement Coping	Efforts to fit in or adapt to the environment	Positive thinking Cognitive restructuring Acceptance Distraction
Disengagement Coping	Oriented away from the stressor or one’s response to it	Avoidance Denial Wishful thinking
Involuntary Engagement		Physiological arousal Rumination
Involuntary Disengagement		Emotional numbing

**Table 2:** Demographics.

Variable	Percent
Race	
Asian	13.16%
African American	2.63%
White	78.95%
Multiple	5.26%
Ethnicity	
Hispanic	5.41%
Not Hispanic	94.59%
Biological sex assigned at birth	
Male	31.58%
Female	68.42%
Gender identity	
Male	31.58%
Female	63.16%
Genderqueer/Gender non-conforming	2.63%
Transgender	2.63%

**Table 3:** Clustering quality metrics for 2-, 3-, and 4-cluster fuzzy solutions.

Number of Clusters	Average Distance	Average Silhouette	F(U)	Fc(U)	D(U)	Dc(U)
2	10.218832	0.330802	0.5245	0.049	0.3224	0.6447
3	6.806429	0.246564	0.3538	0.037	0.5357	0.8035
4	5.104196	−1	0.2654	0.026	0.6496	0.8661

Cluster selection was based on maximizing the average silhouette and Fc(U) while minimizing Dc(U).

**Table 4:** Demographics according to cluster membership.

	High-Stress Cluster (Total *n* = 18) *n* (%)	Low-Stress Cluster (Total *n* = 20) *n* (%)	Χ^2^ Statistic df	*p*-Value
Gender Identity			2.40 df = 3	0.49
Male	5 (27.7%)	7 (35%)		
Female	11 (61.1%)	13 (65%)		
Genderqueer/gender diverse	1 (5.5%)	0		
Multiple	1 (5.5%)	0		
Biological Sex			0.22 df = 1	0.63
Male	5 (27.7%)	7 (35%)		
Female	13 (72.2%)	13 (65%)		
Age			1.15 df = 2	0.56
15	1 (5.5%)	0		
17	1 (5.5%)	1 (5%)		
18	16 (88.9%)	19 (95%)		

Chi-square (Χ^2^) analysis was used to examine differences between clusters. df = degrees of freedom.

**Table 5:** Descriptive data: Coping strategies, PedsQL, and PSCI.

Variable	Mean Proportion	Std. Dev.	Minimum	Maximum
Primary Control Engagement Coping	0.17	0.03	0.09	0.23
Secondary Control Engagement Coping	0.26	0.06	0.12	0.40
Disengagement Coping	0.15	0.02	0.11	0.20
Involuntary Engagement	0.24	0.04	0.14	0.33
Involuntary Disengagement	0.18	0.03	0.11	0.24
	Mean	Std. Dev.	Minimum	Maximum
PedsQL Physical Functioning	74.49	20.99	21.87	100
PedsQL Emotional Functioning	62.73	24.05	20.00	100
PedsQL Social Functioning	82.97	18.79	35.00	100
PedsQL School Functioning	71.33	28.44	5.00	100
PedsQL Psychosocial Functioning	72.84	20.72	33.33	100
PedsQL Total Score	73.42	19.42	35.86	100
PSCI	32.00	8.98	16.00	52.00

PSCI = Perceived Severity of Chronic Illness measure; PedsQL = PedsQL measure.

**Table 6:** Pearson partial correlations between PedsQL total score, PSCI, and coping strategies, controlling for patient age, biological sex, and CHC diagnosis category.

Coping Strategies	PedsQL Total Score Correlation	FDR ^a^ *p*-Value	PSCI Correlation	FDR ^a^ *p*-Value
Primary Control Engagement Coping	0.38	0.011	−0.15	0.184
Secondary Control Engagement Coping	0.59	0.001	−0.36	0.065
Disengagement Coping	−0.29	0.039	0.24	0.103
Involuntary Engagement	−0.58	0.001	0.29	0.097
Involuntary Disengagement	−0.47	0.001	0.23	0.103

Analyses used parametric Pearson partial correlations and controlled for age, biological sex assigned at birth, and CHC diagnosis category. ^a^ *p*-values adjusted using Benjamini–Hochberg FDR [45].

**Table 7:** Coping strategies, PSCI scores, and PedsQL scores according to cluster, controlling for age, biological sex, and CHC diagnosis category.

	High-Stress Cluster Mean ± SE *n* = 18	Low-Stress Cluster Mean ± SE *n* = 20	Wald Test	FDR ^a^ *p*-Value
Primary control engagement coping	0.16 ± 0.01	0.18 ± 0.01	1.57	0.170
Secondary control engagement coping	0.24 ± 0.01	0.28 ± 0.01	1.49	0.177
Disengagement coping	0.15 ± 0.01	0.14 ± 0.01	−0.93	0.370
Involuntary engagement	0.26 ± 0.01	0.22 ± 0.01	−2.22	0.060
Involuntary disengagement	0.18 ± 0.01	0.16 ± 0.01	−0.91	0.370
PSCI	36.25 ± 2.25	28.61 ± 2.08	−2.15	0.063
PedsQL Physical Functioning	62.35 ± 5.06	85.66 ± 4.69	2.91	0.014
PedsQL Emotional Functioning	47.2 ± 6.40	77.31 ± 5.94	2.97	0.014
PedsQL Social Functioning	65.62 ± 4.39	98.13 ± 4.07	4.68	0.004
PedsQL School Functioning	54.14 ± 7.41	88.93 ± 6.87	2.97	0.014
PedsQL Psychosocial Functioning	55.65 ± 4.94	88.12 ± 4.58	4.15	0.004
PedsQL Total Score	57.98 ± 4.54	87.26 ± 4.21	4.07	0.004

Analyses used the Wald test and controlled for age, biological sex assigned at birth, and CHC diagnosis category. ^a^ *p*-values adjusted using Benjamini–Hochberg FDR [45].

## Data Availability

The datasets presented in this article are not available because IRB approval for dataset sharing was not obtained.

## Supplementary Materials

The following supporting information can be downloaded at https://www.mdpi.com/article/10.3390/children12070858/s1, Box S1: Twelve Stressors in RSQ for CHC-related stress, Table S1: Mean stressor ratings for all participants and by cluster. Table S2: CHC Diagnosis Category by Cluster.

## Abbreviations

The following abbreviations are used in this manuscript:

CHCs	Chronic health condition(s)
RSQ	Responses to Stress Questionnaire
PSCI	Perceived Severity of Chronic Illness measure
PedsQL	PedsQL measure
HRQOL	Health-related quality of life
