# Supplementary material for: Stress and Coping in Teens with Chronic Physical Health Conditions: A Cross-Sectional Study
Source: Children (Basel). 2025 Jun 28;12(7):858. doi: 10.3390/children12070858 (PMC12293125; doi:10.3390/children12070858)
Supplement: Supplementary file 1 [file children-12-00858-s001.zip › children-3674518-supplementary.pdf]

Box S1 – Twelve stressors assessed in RSQ for CHC-related stress

Stressor 1	Feeling different from other people because of my chronic health condition
Stressor 2	Not being able to do things other people my age can do because of my chronic health condition
Stressor 3	Feeling like no one else understands what it's like to have a chronic health condition
Stressor 4	Having to take medications for my chronic health condition
Stressor 5	Having to explain my chronic health condition to people
Stressor 6	Being afraid that something bad might happen with my chronic health condition
Stressor 7	Disagreements or arguments with my parents about my chronic health condition
Stressor 8	Managing my chronic health condition when I'm away from home
Stressor 9	Having to go to the clinic or the hospital because of my chronic health condition
Stressor 10	Worrying that my chronic health condition will get worse
Stressor 11	Having problems or complications from my chronic health condition when I'm around other people
Stressor 12	Having difficulty keeping up with school and / or work because of my chronic health condition

Table S1. Mean stressor ratings for all participants and by cluster

Variable	**Statistic**	**Total N = 38**	**High Stress cluster (Total N = 18)**	**Low Stress cluster (Total N = 20)**	**p-value**
**Stressor 1**	Mean	2.158	2.944	1.45	
	Standard Deviation	1.05	0.72	0.75	0.001
**Stressor 2**	Mean	2.395	3.111	1.75	
	Standard Deviation	1.02	0.83	0.71	0.001
**Stressor 3**	Mean	2.105	2.667	1.6	
	Standard Deviation	0.92	0.68	0.82	0.001
**Stressor 4**	Mean	2.474	2.778	2.2	
	Standard Deviation	1.03	1.11	0.89	0.058
**Stressor 5**	Mean	2.324	2.778	1.895	
	Standard Deviation	0.91	0.80	0.80	0.001
**Stressor 6**	Mean	2.605	3.056	2.2	
	Standard Deviation	0.97	0.80	0.95	0.007
**Stressor 7**	Mean	2.026	2.556	1.55	
	Standard Deviation	0.97	0.92	0.75	0.001
**Stressor 8**	Mean	2.622	3.235	2.1	
	Standard Deviation	1.08	0.90	0.96	0.001
**Stressor 9**	Mean	2.026	2.667	1.45	
	Standard Deviation	0.97	0.90	0.60	0.001
**Stressor 10**	Mean	2.316	2.944	1.75	
	Standard Deviation	1.09	0.80	1.01	0.001
**Stressor 11**	Mean	2.132	2.667	1.65	
	Standard Deviation	0.99	1.02	0.67	0.001
**Stressor 12**	Mean	1.737	2.444	1.10	
	Standard Deviation	0.97	0.98	0.30	0.001

Table S2. CHC Diagnosis Category by Cluster

	High stress cluster (Total N=18)	Low stress cluster (Total N=20)	X^2 Statistic df	p-value
			19.03 df=5	0.002
**Asthma**	4	10		
**Food allergy**	0	5		
**GI conditions**	2	4		
**Multiple conditions**	3	1		
**Other conditions**	7	0		
**Type 1 diabetes**	2	0		
